# Acute kidney injury and long-term renal effects of alectinib in anaplastic lymphoma kinase-positive non-small cell lung carcinoma: a case report

**DOI:** 10.1186/s13256-022-03532-2

**Published:** 2022-09-29

**Authors:** Marco van Londen, Elizabeth Roosma, Stefanie Vogels, John W. G. van Putten, Wilbert M. T. Janssen

**Affiliations:** 1 Department of Internal Medicine, Martini Hospital Groningen, Van Swietenplein 1, 9728 NT Groningen, The Netherlands; 2 Department of Pulmonology, Martini Hospital Groningen, Van Swietenplein 1, 9728 NT Groningen, The Netherlands

**Keywords:** Acute kidney injury, Alectinib, Anaplastic lymphoma kinase, Tubular toxicity, Case report

## Abstract

**Background:**

Targeted therapy with anaplastic lymphoma kinase inhibitor alectinib has become standard therapy for selected patients with non-small cell lung carcinoma. Few data are available on the renal effects of alectinib. We report on a case of acute kidney injury in a patient using alectinib for less than 2 weeks and on serum sodium and creatinine during long-term use of alectinib.

**Case presentation:**

A 70-year-old Asian woman was diagnosed with metastasized non-small cell lung carcinoma (cT4N3M1c, stage IV) with echinoderm microtubule-associated protein-like 4 and anaplastic lymphoma kinase gene rearrangement and received alectinib, in two daily doses of 600 mg. Eleven days after the initiation of therapy, she was seen at the emergency department with acute kidney injury. Renal biopsy showed lesions in the proximal tubular epithelial cells. Nine days after alectinib cessation, renal function recovered quickly and reintroduction of alectinib in a reduced dose was tolerated, while withholding metformin, enalapril, and naproxen. In seven other patients, data on estimated glomerular filtration rate showed decreased kidney function at 3 months with stabilization at 6 months. Serum sodium at 3 months increased during alectinib treatment and increased further at 6 months.

**Conclusions:**

Our data suggest direct or indirect toxic (proximal) tubulopathy due to alectinib with a good prognosis after cessation. Adverse acute renal effects of alectinib may be prevented by avoiding other medication influencing renal hemodynamics, in particular nonsteroidal anti-inflammatory drugs. Without these co-medications, alectinib could be reintroduced in our patient.

## Introduction

Targeted therapy has become standard therapy in patients with non-small cell lung carcinoma (NSCLC). Alectinib is a second-generation anaplastic lymphoma kinase (ALK; CD246) inhibitor, used in patients with NSCLC with the echinoderm microtubule-associated protein-like 4–anaplastic lymphoma kinase (EML4–ALK) gene rearrangement [1]. Alectinib has shown superior response rates compared with chemotherapy and the first-generation ALK inhibitor crizotinib. Although adverse renal effects have been reported for other ALK inhibitors [2, 3], few data are available for alectinib. Here, we report on a case of acute kidney injury in a patient using alectinib for 11 days, and on serum sodium and kidney function in patients with ALK-positive NSCLC.

## Case report

**Fig. 1:**
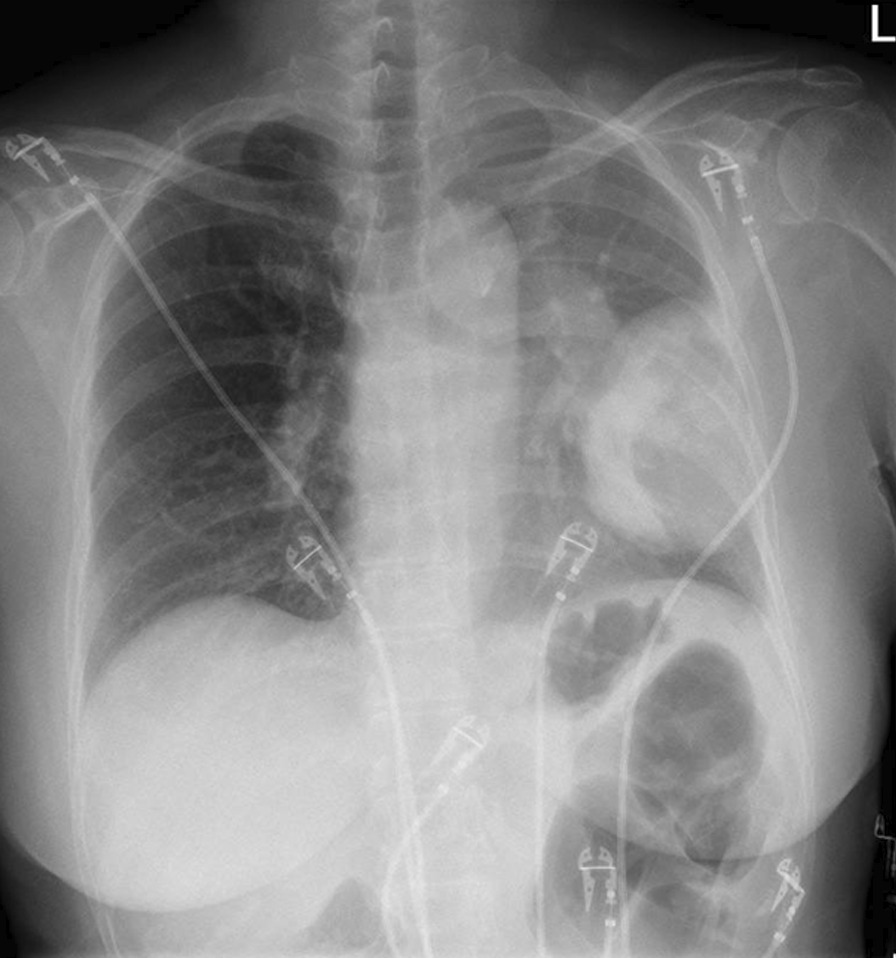
Chest radiograph of patient on diagnosis of non-small cell lung carcinoma

A 70-year-old Asian woman presented to the emergency ward with acute kidney injury after having been diagnosed recently with a non-small cell lung carcinoma (NSCLC). She had a history of hypertension, hypercholesterolemia, and type 2 diabetes for which she used metformin and enalapril. She was of Asian descent, born in Thailand, and lived in the Netherlands for approximately 20 years. For the largest part of her life, she was a stay-at-home mother of two children and had no known occupational exposure to toxins or chemicals. She had a negative family history for pulmonary disease or kidney diseases, never smoked, and consumed no alcohol. She was analyzed for lung cancer after a large lung mass was seen on a chest X-ray carried out for complaints of dyspnea (Fig. 1). The fluorodeoxyglucose (^18^F-FDG) positron emission tomography (PET) with low-dose computed tomography (CT) scan showed an FDG-avid mass in the left lower quadrant of 11 × 6.8 cm with multiple small FDG-avid lesions in all lung quadrants, pathological lymphadenopathy subcarinal in the hili and in the mediastinum, and multiple FDG-avid liver and bone lesions all suspected of being metastases. A bronchoscopy showed an exophytic tumor in the left main bronchus extending to the left upper lobe closing of the main bronchus as well as a tumor in the left lower lobe. The bronchoscopy samples showed NSCLC with *EML4*–*ALK* rearrangement. A multidisciplinary team (pulmonary oncologists, surgeons, nurses, pathologist, radiologist, and nuclear radiologist) discussed the patient in accordance with local guidelines and came to a diagnosis of NSCLC, cT4N3M1c, stage IV with *EML4*–*ALK* rearrangement. She started treatment with alectinib 600 mg twice daily and used naproxen for pain complaints from her bone metastases. Prior to start of alectinib, she had a blood pressure of 143/69 mmHg, serum creatinine of 69 µmol/L [0.78 mg/dL; estimated glomerular filtration rate (eGFR) of 77 mL/min/1.73 m^2^].

At presentation, 11 days after initiation of alectinib, she had complaints of reduced appetite, vomiting, and oliguria. On examination, we found a blood pressure of 120/68 mmHg with a pulse of 81 beats per minute. She had capillary refill time of 4–5 seconds and reduced skin turgor. Her temperature was 36.1 °C (97 °F). Heart auscultation revealed normal hearts sounds without murmurs. Lung auscultation revealed sharpened breath sounds, but no crackles or rhonchi. Examination of the abdomen showed no visible pathology, normal bowel sounds, and no signs of acute abdominal or surgical pathology. Basic neurological examination revealed no impaired mental status, no cranial nerve pathology, normal gait and coordination, and no abnormalities on motor and sensory examination. Further physical examination revealed no other abnormalities. A chest X-ray showed a reduction of the lung cancer mass compared with the initial staging imaging. Laboratory evaluation revealed a creatinine level of 424 µmol/L (4.79 mg/dL; eGFR 8 mL/min/1.73 m^2^), and a urea level of 15.3 mmol/L (91.8 mg/dL). Other lab results showed anemia (hemoglobin 6.6 mmol/L, 10.6 g/dL), thrombocytosis (691 × 10^9^/L), and leukocytosis (14.9 × 10^9^/L). She had hyponatremia (127 mmol/L; 127 mEq/L), hyperkalemia (6.0 mmol/L, 6 mEq/L), and hypercalcemia (2.61 mmol/L, 10.46 mg/dL) that was normal when corrected for albumin (31 g/L, 3.1 g/dL). She had normal alanine transaminase and aspartate transaminase, but alkaline phosphatase and gamma-glutamyl transferase were slightly above the upper limit of normal (170 U/L and 91 U/L, respectively). The C-reactive protein was slightly above the upper limit of normal (7 mg/L, 0.7 mg/dL). Her glucose at presentation was 8.3 mmol/L (149.5 mg/dL). Urine analysis showed leukocyturia (66/µL) without hematuria and no bacteriuria. Urinary sodium concentration was 73 mEq/L (167.9 mg/dL) and the fractional excretion of sodium was 11%, suggesting renal tubular etiology. Ultrasound of the kidneys showed no abnormalities. No serologic or microbiological tests were performed.

**Fig. 2:**
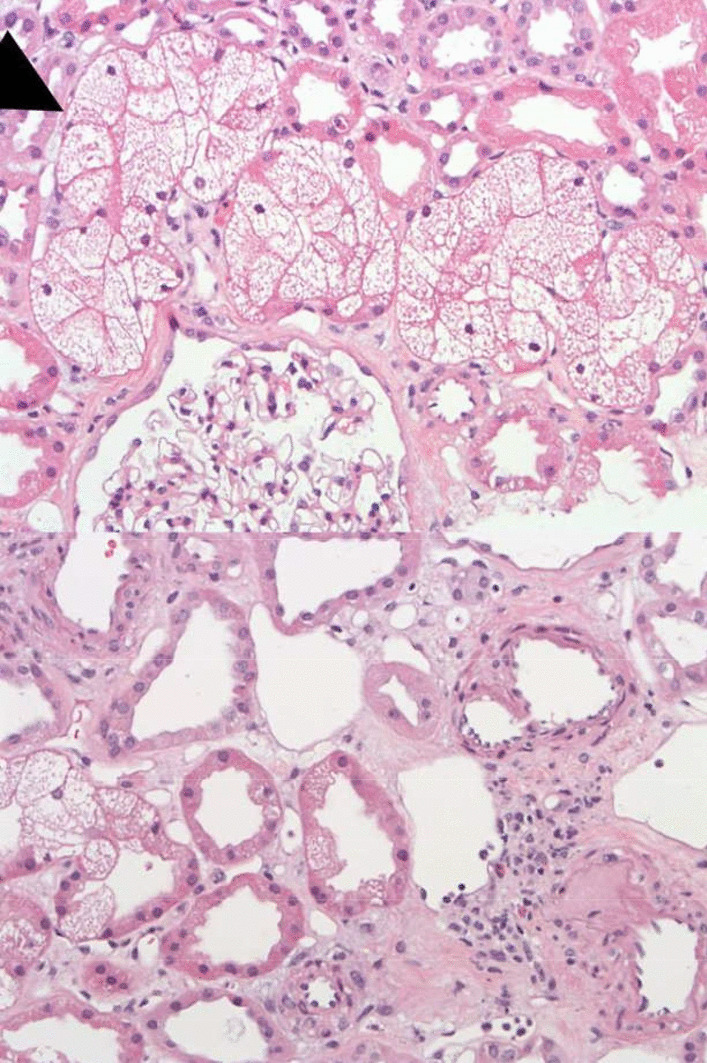
Tubular toxicity in a patient on alectinib. Light microscopy reveals massive vacuolar alterations of the cytoplasm of proximal tubular epithelial cells, representing tubular toxicity. No glomerular pathology or vascular changes were seen. Immunofluorescence studies were negative, nor were signs seen of interstitial nephritis

The patient was admitted to the pulmonology ward and received sodium chloride 0.9% fluid therapy, 1 L in 4 hours followed by 3 L/24 hours. All medication was discontinued. For pain complaints, acetaminophen was prescribed (four times 1000 mg daily) and fentanyl was started, as both transdermal patch (12.5 µg/hour) and tablets for sublingual that the patient used at her own discretion with a maximum of 200 µg per day. For nausea, metoclopramide 10 mg tablets were started, taken at the patient’s discretion with a maximum of three times daily. Because of constipation, macrogol and lactulose were started, and during the hospital admission a sodium phosphate enema was administered. Temazepam 10 mg was used at the patient’s discretion before sleeping, with a maximum of once daily. Nadroparin 2850 IU was started to prevent venous thromboembolisms. During the admission, insulin aspart was given for hyperglycemia when necessary (0–10 IE based on glucose levels and intake). The patient’s diuresis during the first 24 hours was 1860 mL, which further suggested tubulopathy as a possible cause of her acute kidney injury. After 2 days of fluid therapy, there was no improvement of renal function, and a biopsy was performed. Light microscopy revealed massive vacuolar alterations of the cytoplasm of proximal tubular epithelial cells (Fig. 2), representing proximal tubular toxicity. Glomerular, interstitial, and vascular structures were normal. No tubular cell sloughing, necrosis, cast formation, or interstitial edema was seen.

On the fourth day, intravenous prednisolone 40 mg daily was started for 7 days. In the days thereafter, renal function recovered rapidly, blood pressure rose to 148/80 mmHg, and the patient was discharged 9 days after admission with an eGFR of 70 mL/min/1.73 m^2^. Alectinib was reintroduced 1 week after discharge at 300 mg twice daily and 2 weeks later increased to 450 mg twice daily without decrease in kidney function until she died 2 months after discharge. Metformin, enalapril, and naproxen were withheld completely during the follow-up. During follow-up, the patient continued using metoclopramide and temazepam when necessary, and the fentanyl dose was increased step by step to a dose of 50 µg/hour in the weeks leading up to her death. In the palliative phase, no other medication was started. In a final staging ^18^F-FDG PET scan before her death, a reduction of size and activity of the primary tumor and the pathological lymph nodes was seen, but the patient had developed new pathological vertebral and pelvic fractures. She died of complications of her non-small cell lung carcinoma 2 months after discharge. No autopsy was performed.

## Long-term renal effects of alectinib

To obtain more insight into the renal effects of alectinib, we collected the renal laboratory values of seven other patients treated with alectinib for at least 3 months. In seven other patients treated with alectinib for 3 months, serum sodium values slightly increased during treatment [from median 140 (135–142) to 142 (139–145) mmol/L, Table 1]. At 6 months, four patients showed a further increase in serum sodium [median 144 (141–146) mmol/L]. Serum creatinine rose in all seven patients at 3 months [from median 63 (56–89) to 73 (64–101) μmol/L, Table 1], with a further rise in three patients and a decline in two patients at 6 months, suggesting a decline at 3 months with a stabilization at 6 months (Table 1).

**Table 1:** Long-term renal effects of alectinib

	Baseline (*N* = 7)	3 months (*N* = 7)	6 months (*N* = 5)
Sodium, mmol/L	140 (135–142)	142 (139–145)	144 (141–146)
Creatinine, μmol/L	63 (56–89)	73 (64–101)	76 (66–103)
eGFR, mL/min/1.73 m^2^	89 (60–121)	79 (52–111)	88 (50–113)

Data are presented as median (range)

eGFR, estimated Glomerular Filtration Rate; N, number

## Discussion and conclusions

Here, we report on acute kidney injury and long-term renal effects related to the ALK inhibitor alectinib. We describe a 70-year-old woman with metastasized non-small cell lung carcinoma (cT4N3M1c, stage IV) with *EML4*–*ALK* gene rearrangement who received alectinib and was seen 11 days later at the emergency department with an acute kidney injury and lesions in the proximal tubular epithelial cells on kidney biopsy. We show that reintroduction of alectinib at a reduced dose was possible, when withholding medication that interferes with renal hemodynamics.

Acute kidney injury is reported as an adverse effect of alectinib [4]. A previous case report showed renal impairment shortly after the start of alectinib [5], suggesting a direct toxic effect and tubular damage, without biopsy data. Another case reported acute renal function loss without a possible mechanism or with slowly progressive kidney insufficiency with nephritis and glomerular podocyte damage on biopsy [6]. In our case, we demonstrate a swollen proximal tubular epithelium without signs of acute tubular necrosis or nephritis, indicating toxic tubulopathy due to alectinib. The biopsy showed only minimal interstitial inflammation. We therefore attribute the recovery of renal function to cessation of alectinib rather than to intravenous prednisolone, which we started on the basis of the report of Nagai *et al*. [6]. A full-dose rechallenge with alectinib might cause a decrease of kidney function. We therefore reintroduced alectinib at a low dose after discharge and increased the dose when serum creatinine levels remained stable. We withheld nonsteroidal anti-inflammatory drugs (NSAIDs) and renin–angiotensin–aldosterone system (RAAS) blockers during reintroduction of alectinib as a precautionary measure.

An increase in weight and edema are reported as very frequent (> 10%) adverse events in the long-term use of alectinib [4, 7], suggesting water and sodium retention. We found serum sodium and serum creatinine to rise after 3-month use of alectinib. Data on another ALK inhibitor (crizotinib) also showed elevation of serum creatinine [3]. A study by Meijer-Schaap *et al*. on the renal hemodynamic effects of crizotinib may add data on the renal mechanism of these effects. Patients with crizotinib had an extensively increased filtration fraction, a measure of glomerular pressure [2]. This indicates that the ALK inhibitor crizotinib may increase postglomerular vascular resistance, with hyperfiltration on the one hand and a lower tubular blood flow on the other as possible results. This combination mimics a sodium-retaining dehydrated state with elevations in interstitial and papillary osmolality in long-term use of ALK inhibitors. Renal medullary concentrating mechanisms in the presence of hyperfiltration of water, sodium, and creatinine retain water in combination with net tubular reabsorption of sodium and creatinine, leading to a rise in serum sodium [8] and creatinine [2, 9]. Our data can thus be explained by a potent postglomerular vasoconstrictive effect of alectinib as seen with crizotinib. Such a postglomerular vasoconstrictive effect can also contribute to susceptibility to tubular damage and acute kidney injury.

Our patient used a nonsteroidal anti-inflammatory drug (NSAID) and an angiotensin-converting enzyme inhibitor (ACE inhibitor) on admission with acute kidney injury. As discussed above, we suggest a strong postglomerular vasoconstrictive effect of alectinib to induce a lower tubular blood flow that compromises tubular oxygen supply. NSAID-induced preglomerular vasoconstriction will enhance this effect. Reintroduction of alectinib was possible when withholding NSAIDs. In patients starting on alectinib while using NSAIDs, renal function should be monitored carefully if alternative pain medication is not possible. In the setting of acute kidney injury, we also stopped ACE inhibition and did not start it after recovery. Theoretically, the use of ACE inhibition might be favorable by counteracting the postglomerular vasoconstrictive action of alectinib, but our case did not have the data available to test this hypothesis. This needs further investigation.

## Conclusion

We conclude that alectinib may have potent postglomerular vasoconstrictive effects with consequent renal tubular effects, which may lead to a rise in serum sodium and creatinine levels in the long term and in some patients to severe tubular toxicity and acute kidney injury. Medication that interferes with renal hemodynamics, in particular NSAIDs, should be avoided. Reintroduction of reduced dose of alectinib is a viable strategy when withholding such medication.

## Data Availability

Not applicable.

## Abbreviations

NSCLC: Non-small cell lung carcinoma
ALK: Anaplastic lymphoma kinase
eGFR: Estimated glomerular filtration rate
NSAID: Nonsteroidal anti-inflammatory drugs
ACE: Angiotensin-converting enzyme
RAAS: Renin–angiotensin–aldosterone system
